# Improved performance with automatic sound management 3 in the MED-EL SONNET 2 cochlear implant audio processor

**DOI:** 10.1371/journal.pone.0274446

**Published:** 2022-09-15

**Authors:** Anja Kurz, Kristen Rak, Rudolf Hagen

**Affiliations:** Department of Otorhinolaryngology, Plastic, Aesthetic and Reconstructive Head and Neck Surgery, Comprehensive Hearing Center, University Hospital of Würzburg, Würzburg, Germany; Hannover Medical School: Medizinische Hochschule Hannover, GERMANY

## Abstract

**Objectives:**

The SONNET 2 audio processor features ambient noise reduction (ANR), transient-noise reduction (TNR), and adaptive intelligence (AI). The primary aim of this study was to evaluate if using these features improves speech perception in noise, subjective listening effort, and sound quality.

**Design:**

In this prospective longitudinal study, twenty adult SONNET users were fitted with the SONNET 2 audio processor, configured either as a default SONNET (no ANR/TNR/AI), with mild ANR/TNR, with strong ANR/TNR, with mild AI, and with strong AI. Speech perception in noise was assessed in speech and stationary noise from the front (S0N0); speech, stationary noise, and transient noise from the front (S0N0T0); and speech from the front in spatially-distributed stationary noise (S0N±45N±135). Listening effort, subjective sound quality, and device/setup preference were assessed.

**Results:**

In the S0N0 setup, speech perception in noise was significantly better with the SONNET 2 when using ANR/TNR in the mild setup than with the SONNET or the SONNET 2 in the default SONNET configuration. In the S0N±45N±135 setup, speech understanding was significantly better in all four SONNET 2 configurations than with the SONNET or the SONNET 2 in the default SONNET configuration (a 1.26–2.55 dB SRT80 benefit). Subjects tolerated consistently lower signal-to-noise values with the SONNET 2 configurations using ANR/TNR than with the default SONNET configuration in all listening effort categories. All SONNET 2 configurations using ANR/TNR were preferred and better rated in speech in stationary and/or transient noise compared to the default SONNET configuration. Sound quality and pleasantness were better in those SONNET 2 configurations. Subjects strongly preferred the SONNET 2 configurations over the SONNET configuration.

**Conclusions:**

The new front-end features implemented in the SONNET 2 audio processor objectively improve speech perception in noise. Subjects preferred the SONNET 2, over the SONNET, in the presence of stationary and transient noise.

## Introduction

While cochlear implant (CI) users’ ability to understanding speech has continuously improved over the last two decades [1], it is still significantly more susceptible to background noise compared to that of people with normal hearing [2, 3]. CI users may achieve ceiling-level performance of sentence recognition in quiet [4], however, they need a high signal-to-noise ratio (SNR) to understand speech in the presence of background noise [5]. To reach a 50% speech reception threshold (SRT), SNRs of at least +5dB up to +25dB are necessary [1, 6–9]. For CI users, difficulties in understanding speech are even more pronounced in the presence of fluctuating noise than in stationary noise or in noise with low temporal variations. This results in a considerable increase in listening effort and an overall loss in quality of life [6, 7, 10–12]. Consequently, reducing the amount of noise is an important and enduring issue in the CI field.

Noise reduction algorithms have been implemented in the front-end signal processing of CI audio processors by all major CI manufacturers. MED-EL (Innsbruck, Austria) introduced wind-noise reduction and fixed and adaptive directional processing (i.e., beamforming) with the SONNET audio processor in 2014. This technology enabled significantly improved speech performance in background noise compared to the predecessor system [13–17]. The recently launched SONNET 2 audio processor features two further noise reduction algorithms: ambient noise reduction (ANR) and transient noise reduction (TNR). ANR aims to reduce noise in a sound signal where the target signal (e.g., speech) is contaminated with stationary or quasi-stationary background noise (with no, or comparably slow, temporal variations). Examples for such noise signals are the ones that emanate from a ventilating system or vehicle such as a car, airplane, or train [18–20]. In the SONNET 2, ANR is available either with moderate noise suppression (“mild” setting) or with stronger noise suppression (“strong” setting). The “strong” setting provides larger noise reduction at the cost of potential target signal distortions.

TNR aims to decrease the amplitude of short, impulsive signal bursts in the sound signal. These transient sounds are characterized by a rapid onset to the peak in sound pressure level, a fast decay, and a short duration. The peak sound pressure level of the transient noise is well above the average sound pressure level [21]. Transient sounds can be loud (slamming doors, hammer blows, or clanking of dishes) or soft (such as the clicking of a pen or typing on a computer keyboard) [22]. They have been characterized as distracting and are a frequent source of complaint for people with impaired hearing [23]. In the SONNET 2, TNR is implemented with either moderate (“mild” mode) or stronger suppression (“strong” mode).

Both stationary-noise as well as transient-noise reduction systems have been realized in currently marketed state-of-the-art CI systems. Stationary-noise reduction is a feature of ClearVoice by Advanced Bionics (Stäfa, Switzerland), SNR-NR as part of Smart Sound iQ by Cochlear Ltd (Macquarie University, Australia), and VoiceTrack by Oticon Medical/Neurelec (Vallauris, France). Other than at MED-EL, a dedicated transient-noise reduction algorithm is presently only available by Advanced Bionics, as a component of ClearVoice based on an original Phonak technology named SoundRelax.

The growing number of front-end sound processing technologies implemented in CI systems can lead to uncertainty when to change programs. In real life, few users change from their everyday settings to try to avoid noisy situations [24], which may prevent them from achieving best hearing performance [20]. To this end, signal classifiers have been developed that can detect acoustic situations and switch between programs and pre-processing algorithms [25]. In the SONNET 2, a signal-classification-based system named Adaptive Intelligence (AI) discriminates five signal classes: Speech, Speech in Noise, Noise, Music, and Quiet. The purpose of the signal classification is to estimate the exposure time of the CI user in a certain acoustic environment and to enable an automatic selection of signal processing algorithms, e.g. directionality and/or noise reduction, depending on the detected signal class, as shown in Table 1 (see [17]), for an in-depth investigation of the directionality legacy feature). As can also be seen from Table 1, only ANR and TNR are activated or deactivated automatically in AI mild mode. The directionality remains in fixed beamforming mode, whereby the directional characteristic is tuned in such way as to approximate the natural directionality provided by the human pinna (Natural mode). ANR is further active in all signal classes, except music, whereas TNR is only active if noise is classified, either in the presence of only noise or in noise and speech. The AI strong mode provides a more intervening signal processing in that directionality is also switched between omnidirectional and adaptive directional, and ANR and TNR are activated in the strong modes. The adaptive directional mode is an adaptive beamformer (ABF) which adjusts its directional characteristic to maximally reduce the noise, i.e., it adapts to the direction and spectral characteristic of the noise sources. Note that in both AI modes, in the "Quiet" signal class, the system remains in the previous state, according to the previously detected signal class.

**Table 1:** Adaptive intelligence (AI) activation scheme.

AI	Signal Class	Directionality	ANR	TNR
**Mild**	Quiet	Previous state maintained		
	Speech	Natural	Mild	Off
	Speech in noise	Natural	Mild	Mild
	Noise	Natural	Mild	Mild
	Music	Natural	Off	Off
**Strong**	Quiet	Previous state maintained		
	Speech	Adaptive	Strong	Off
	Speech in noise	Adaptive	Strong	Strong
	Noise	Adaptive	Strong	Strong
	Music	Omni	Off	Off

Note: if “Quiet” is classified, the system remains in the previous state, according to the previously detected signal class. ANR = Ambient noise reduction, TNR = transient noise reduction

Automatic signal classification technologies have been recently introduced in some commercially available CI systems. Cochlear Ltd’s SmartSound iQ has implemented the automatic signal classifier SCAN in its currently marketed audio processors. Results revealed objective and subjective improvements in speech understanding for both adult and paediatric users [4, 20, 26, 27]. Similar systems are also promoted by Advanced Bionics (AutoSound™ OS) and Oticon Medical/Neurelec (Coordinated Adaptive Processing), but so far, no publicly available documentation is available on these products.

The primary aim of the present study was to evaluate the added ANR, TNR, and AI capabilities of the new SONNET 2 on speech understanding in noise by comparing them to the SONNET audio processor and to the SONNET 2 in the default SONNET configuration. The secondary aim was to access the subjective aspects of using the new device and configurations. To this end, listening effort, sound quality, and satisfaction with audio processor were collected and analysed.

## Materials and methods

The study was approved by the ethics committee of the University of Würzburg, Germany and conducted following the Declaration of Helsinki. Written informed consent was obtained from all subjects prior to any study-specific procedure.

### Participants

To be included in the study, participants had to meet all of the following inclusion criteria: 1) be at least 18 years old, 2) have ≥ 6 months of experience with a MED-EL CI (C40+ or later model), 3) have ≥ 6 months experience using the MED-EL SONNET audio processor, 4) have postlingual onset of bilateral severe to profound sensory-neural hearing loss, 5) be a unilateral CI users, 6) have a minimum of 10 active electrodes on their implanted array, 7) score ≥40% speech recognition on the Freiburg Monosyllables Test in quiet at 65dB SPL (at the last time tested), 8) be fluent in German (the language of the test centre), and 9) have signed and dated their informed consent before the start of any study-specific procedure.

### Audio processors

The SONNET 2 processor was investigated in five configurations, as shown in Table 2. Configuration S2.Sonnet resembles a SONNET processor, which is the predecessor of the SONNET 2. The S2.Mild, S2.Strong, S2.AImild, and S2.AIstrong configurations represent the SONNET 2 processor with different combinations of ANR, TNR, and AI, together with certain settings of the already established front-end features wind-noise reduction (WNR), directionality, and automatic gain control (AGC). For details on AGC, please see Stöbich et al. [28]. S2.AImild is the clinical default setting of the SONNET 2 processor. In addition to performance with the SONNET 2 processor, the subjects’ SONNET processors were also tested as a baseline in the clinical default setting. Technical integrity was checked and microphone covers were replaced, if needed. Configurations are shown in Table 2.

**Table 2:** Audio processors and tested audio processor configurations.

Audio Processor	Configuration	AI	WNR	Direct.	AGC Compression	AGC Sensitivity	ANR	TNR
**SONNET**	(clinical default)	N/A	Mild	NAT	3:1	75%	N/A	N/A
**SONNET 2**	S2.Sonnet	Off	Mild	NAT	3:1	75%	N/A	N/A
**SONNET 2**	S2.Mild	Off	Mild	NAT	3:1	75%	Mild	Mild
**SONNET 2**	S2.Strong	Off	Mild	NAT	3:1	75%	Strong	Strong
**SONNET 2**	S2.AImild (clinical default)	Mild	Mild	Auto (NAT)	3:1	75%	Auto (Off-Mild)	Auto (Off-Mild)
**SONNET 2**	S2.AIstrong	Strong	Strong	Auto (OMNI-ABF)	3:1	75%	Auto (Off-Strong)	Auto (Off-Strong)

ABF = adaptive beamformer, AGC = automatic gain control, AI = adaptive intelligence, ANR = Ambient noise reduction, Auto = automatic switching of directionality and noise suppression, Direct. = directionality, NAT = fixed (‘natural’) beamformer, OMNI = omnidirectional, TNR = transient noise reduction, WNR = wind noise reduction

### Study design

This was a prospective longitudinal monocentric study conducted at the Universitätsklinikum Würzburg, Germany, with each subject acting as his/her own control. Subjects made five visits to the clinic and completed one test session in each of the visits. See Table 3 for an overview of the study schedule and the different configurations tested at each visit. The order in which the configurations were tested within each visit was randomized between subjects (Table 3).

**Table 3:** Testing schedule with fitting configurations.

Visit	Tested	Configurations used
**1**	Speech in quiet, APSQ, HISQUI, SSQ12	SONNET, S2.Sonnet, S2.Mild, S2.Strong, S2.AImild (clinical default), S2.AIstrong
**2**	Speech in noise (S0N0, S0N0T0), HISQUI, SSQ12	SONNET, S2.Sonnet, S2.Mild, S2.Strong, S2.AImild (clinical default), S2.AIstrong
**3**	Speech in noise (S0N±45N±135), APSQ, HISQUI, SSQ12	SONNET, S2.Sonnet, S2.Mild, S2.Strong, S2.AImild (clinical default), S2.AIstrong
**4**	ACALES, subjective sound quality ratings, HISQUI, SSQ12	SONNET, S2.Sonnet, S2.Mild, S2.Strong
**5**	ACALES, subjective sound quality ratings, HISQUI, SSQ12	SONNET, S2.Sonnet, S2.Mild, S2.Strong

*Note*: S0N0T0 was tested only in the S2.Sonnet, S2.Mild, and S2.Strong configurations. ACALES = Adaptive Categorical Listening Effort Scaling, AI = adaptive intelligence, APSQ = Audio Processor Satisfaction Questionnaire, HISQUI = Hearing Implant Sound Quality Index, SSQ12 = Speech, Spatial and Qualities of Hearing scale

At the first visit, subjects were tested using their SONNET and answered the questionnaires as regards their experience with the SONNET (not the SONNET 2). They were then upgraded with a SONNET 2. Subject’s old SONNET map was used as a base for the new SONNET 2 fitting. For the new fitting, the thresholds and MCLs were taken unchanged from their original fitting and the new signal processing (ANR, TNR, AI) were activated according to the test plan. MAESTRO software suite 8 and the MAX programming interface were used for fitting.

Between each of the visits, in four take-home periods (each 1–3 weeks long), subjects used one of the following SONNET 2 configurations in an order that was randomized between subjects: S2.Mild, S2.Strong, S2.AImild, and S2.AIstrong. Subjects were asked to predominantly use the SONNET 2 configuration; however, if they found it unbearable, they could revert to using the default SONNET 1 configuration (i.e., BF = NAT & WNR = Mild). Care was taken to confirm usage of the SONNET 2 configurations via the datalogging feature of the processor.

### Test setups

**Fig 1:**
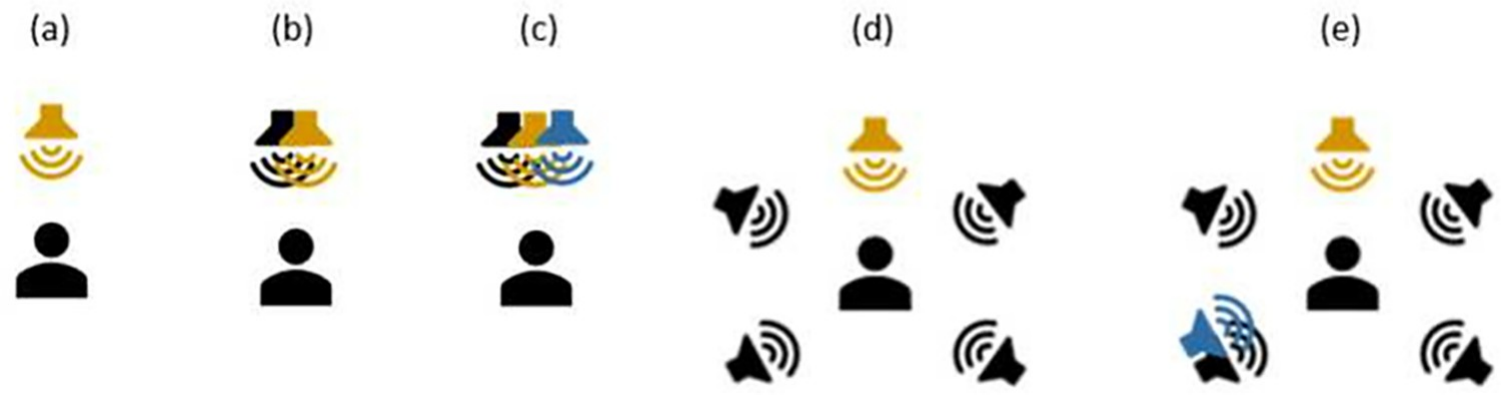
Speech test setups for (a) speech or music testing in quiet (S0 or M0), (b) speech in noise S0N0, (c) speech in noise S0N0T0, (d) speech in noise S0N±45N±135, and (e) speech in noise S0N±45N±135T135 (note: transient noise presented ipsilaterally to ear with the CI). Orange speaker icons indicate target signal (speech or music), black speaker icons indicate stationary noise, and the blue speaker icons indicate transient noise.

- Speech perception tests and subjective ratings were performed in five different test setups: S0 or M0: Speech or music (presented from the front).

- S0N0: Speech in stationary noise (both presented from the front).
- S0N0T0: Speech in stationary and transient noise (all presented from the front).
- S0N±45N±135: Speech in stationary noise (speech presented from the front, stationary noise presented from the sides, noise speakers presented uncorrelated noise).
- S0N±45N±135T135: Speech in stationary and transient noise (speech presented from the front, stationary noise presented from the sides, and transient noise presented ipsilaterally to the CI-side at either +135° or -135°) (Fig 1).

### Speech understanding tests

Speech perception in quiet was evaluated with the Freiburg Monosyllables Test [29] in the S0 setup (see Fig 1). Speech perception in noise was assessed with the Oldenburg Sentence Test (OLSA) [30] for 80% correct word recognition in noise (SRT80 in dB SNR). SRT80 was used because this results in larger SNR values, which then limit possible speech distortions introduced by the ANR if the SNR approaches low levels, e.g., around 0dB, in high-performing users. The stationary noise-level was fixed and presented continuously at 60dB sound pressure level (SPL); the noise used was the OLSA speech-shaped noise. The level of the speech signal was varied according to the OLSA procedure [30]. Transient noise was also presented at a fixed level, which was set to be 3dB higher than the level of the stationary noise. The presentation level of the accumulated noise (stationary and transient noise) in this case was again 60dB SPL. With this setup it was guaranteed that the transient noise was loud enough but did not provoke clipping in the audio processor (due to the large peak-to-average power ratio for the transient noises). The transient noise used was hammering (an iron hammer hammering on iron). The rate was approximately 1–2 strokes per seconds. The stroke duration, measured from the onset of the signal to a signal level below 60dB of the maximum, was approx. 500ms. Due to the short duration of the hammering strokes, the strokes were inherently broadband, thus extended over the entire bandwidth. The speech signal was therefore completely masked during the hammering stroke. Speech perception in noise was tested in three setups: S0N0, S0N0T0, and S0N±45N±135 (see Fig 1). During the testing of subjects who were bimodal users, their hearing aid was switched off and the hearing aid and earmold were left on the ear to act as an additional damping.

### Listening effort

The Adaptive Categorical Listening Effort Scaling (ACALES) test was used to investigate the subjectively perceived listening effort for the S2.Sonnet, S2.Mild, and S2.Strong configurations [31]. Subjects (n = 17) rated the perceived listening effort when listening to OLSA sentences in OLSA speech-shaped noise on a categorical scale ranging from 1 (no effort) to 13 (extreme effort). During the procedure, the SNRs automatically adapted according to the given ratings. It was expected that listening effort would decrease as the SNR increased (and vice versa). Listening effort scaling was performed in two different setups: S0N0 and S0N±45N±135 (see Fig 1). The sound levels were the same as for the speech tests described in the section above, i.e., the noise was presented continuously with 60dB SPL, the speech level was adapted according to the ACALES procedure.

Differences in the results with each configuration were calculated for the three categories low effort (“no effort” to “little effort”, comprising ratings from 1 to 5), moderate effort (“little effort” to “considerable effort”, comprising ratings from 5 to 9), and high effort (“considerable effort” to “extreme effort", comprising ratings from 9 to 13). A positive result (e.g. 2 dB) can then be interpreted in a way that subjects rated listening to a lower (i.e. more difficult) SNR (e.g. 6 dB) with the S2.Mild or S2.Strong configuration as equally strenuous as listening to a higher (i.e. easier) SNR (e.g. 8 dB) with the S2.Sonnet configuration. For determining these SNR differences, a sigmoid-shaped psychometric function was fitted to the rating results for each of the six conditions, i.e., the three processor configurations (S2.Sonnet vs. S2.Mild vs. S2.Strong) and for the two test conditions (S0N0 and S0N±45N±135). If the rating results did not allow a fit, for example, because of inconsistent rating, or the user was not able to exploit the entire range of the effort scale, the subject was excluded from this analysis. Due to this, 3 subjects were excluded.

### Subjective sound quality and preference

Subjects rated their subjective sound quality and preference using a visual analogue scale after listening in six setups: S0, M0, S0N0, S0N±45N±135, S0N0T0, and S0N±45N±135T135 (see Fig 1 for the setups). The sound samples were presented with an acoustic level of 65dB SPL. Subjects ranked preference as well as subjective sound quality for the S2.Sonnet, S2.Mild, and S2.Strong configurations. Concerning subjective sound quality, subjects rated the six setups for four different quality aspects: “Speech and Music Quality”, “Pleasantness”, “Listening Effort”, and “How Disturbing the Noise Is”.

### Questionnaires

The Audio Processor Satisfaction Questionnaire (APSQ) was used to assess user satisfaction with audio processors [32]. Further questionnaires included the Hearing Implant Sound Quality Index (HISQUI), which evaluates subjective sound quality [33], and the Speech, Spatial and Qualities of Hearing scale (SSQ12) for measuring everyday listening abilities and limitations [34].

In addition, subjects used a product-specific questionnaire to grade their preference for one of the four different SONNET 2 configurations (S2.Mild, S2.Strong, S2.AImild, and S2.AIstrong) versus the baseline SONNET configuration (S2.Sonnet) across different listening situations. The questionnaire consisted of the following 14 listening situations::

- talking with one person in a crowded area
- talking with one person in a quiet room
- talking with one person in an area with high reverberation
- talking with one person while the radio or TV is on
- talking with one person next to a busy road
- talking with one person when there is steady noise
- being in an environment where the direction of the sound and the distance to the sound source both play important roles (like on a street)
- being in a cinema or while watching TV in a quiet room
- being outdoors (e.g., while running or cycling)
- being in an environment with short and loud ambient noise
- being in a place where the listening condition changes frequently (e.g., between quiet and noise)
- talking in a place where other people are also talking
- being in a loud environment with more or less steady ambient noise
- listening to music.

For each situation, subjects chose their preference for the new SONNET 2 configuration, the baseline SONNET configuration, no preference (i.e., they were equal), or “not applicable” (“NA”), (i.e., if the subject is never in the specific environment or situation). Subjects could also leave a situation unanswered (“Missing”). Note that during one period the subjects’ audio processor was configured with two programs: the S2.Sonnet (baseline) and one of the four SONNET 2 configurations.

### Data analysis

For analysing results of speech perception tests, configurations were compared using paired-samples t-tests (i.e., t-test) or, depending on the data distribution, Wilcoxon signed-rank tests. The Kolmogorov-Smirnov test and the Shapiro-Wilk test were conducted before to check the data distribution. If both tests confirmed that the data were normally distributed, then parametric statistical methods were applied. Otherwise, non-parametric statistical methods were conducted.

For analysing listening effort tests, for each effort category (low, moderate, high) of the ACALES rating, one-sample t-tests were carried out to test whether the difference in dB between S2.Sonnet, S2.Mild, and S2.Strong were significantly different from zero.

Significance was set to p ≤ 0.05. To control for the problem of multiplicity resulting from multiple pairwise comparisons (i.e., to avoid Type I errors), the Holm-Bonferroni correction method was applied. Missing data were treated as missing values.

IBM SPSS Statistics V24 and V25 (IBM, Armonk, NY, USA) were used for statistical analyses.

## Results

### Participants

Twenty adult unilaterally implanted CI users with bilateral postlingual sensorineural deafness (8 females, 12 males; (14 right ears, 6 left ears) met the inclusion criteria and took part in the study. Some participants were bimodal users, i.e., wore a hearing aid in the contralateral ear. Participants were a mean 48.2 years old (± 16.25 SD; range 17.7–70.4 years) at implantation and a mean 53.7 years old (± 16.95 SD; range 20.4–72.9 years) at enrolment. All subjects had been provided with various MED-EL CIs coupled to either a STANDARD or FLEX-type electrode array: STANDARD arrays are 31.5mm long and have 24 contacts; FLEX-type arrays range from 20–31.5mm long, have added flexibility, and 19 contacts; all are straight lateral wall arrays. All subjects had at least 6 months experience with the SONNET at the time of testing.

### Speech understanding in quiet (S0)

**Fig 2:**
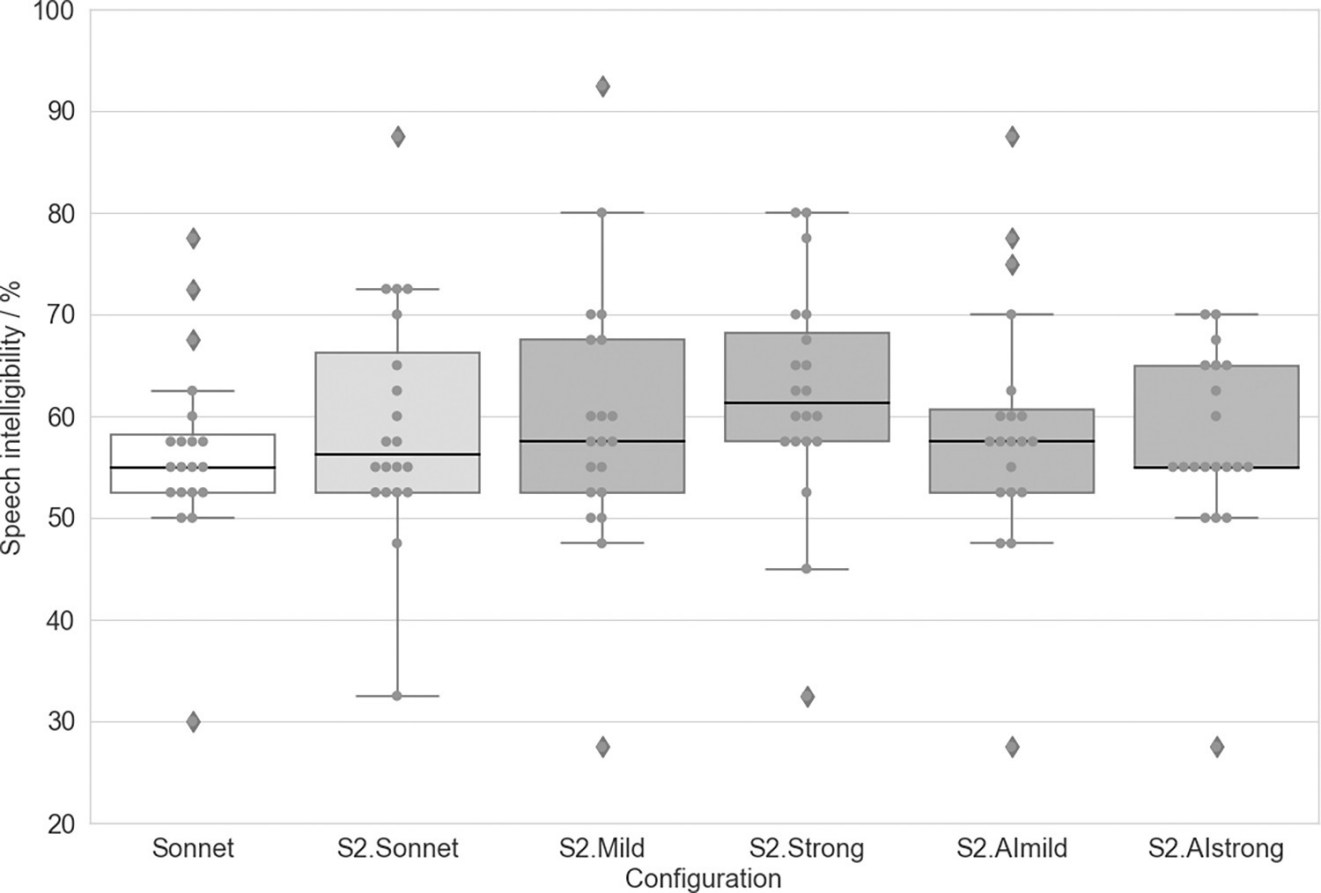
Speech intelligibility in quiet with each configuration. Boxplots show median values as well as first and third quartiles. Whisker bars show minimum and maximum values within 1.5 interquartile ranges below and above the first and third quartile. Diamonds show outliers.

Speech understanding scores for each device/configuration listed in Table 2 are shown in Fig 2 and Table 4.

**Table 4:** Speech understanding scores (in mean, standard deviation, and range) in quiet and speech reception threshold (SRT80) for each device and setup.

	Speech understanding in quiet (% correct)	Speech Understanding in Noise (SRT80 in dB SNR)		
	S0	S0N0	S0N0T0	S0N±45N±135
**SONNET**	56.5% (±9.7%; 30‒77.5%)	6.0 dB (±3.99; 2.34‒20.73)	-	3.08 dB (±3.77; -1.08–15.24)
**S2.Sonnet**	59.4% (±11.7%, 32.5–87.5%)	6.1 dB (±3.18, 2.33–13.89)	6.47 dB (±4.07; 0.12–16.59)	3.23 dB (±3.40, -1.19–13.35)
**S2. Mild**	59.5% (±13.3%; 27.5‒92.5%)	4.67 dB (±3.10; 1.12–12.32)	6.27 dB (±4.69; 0.90–18.29)	1.05 dB (±2.39; -3.57–6.22)
**S2.Strong**	62% (±11.3%; 32.5–80%)	5.71 dB (±3.64; 1.74–15.33)	6.61 dB (±4.35; 1.03–16.70)	1.82 dB (±3.48; -2.58–13.15)
**S2.AImild**	58.8% (±12.5%; 27.5‒87.5%)	4.93 dB (±4.44; 1.25–20.55)	-	0.68 dB (±2.22; -2.42–4.52)
**S2.AIstrong**	57.1% (±9.5%; 27.5‒70.0%)	6.12 dB (±3.45; 2.89–16.76).	-	1.61 dB (±2.36; -2.13–7.96)

Pairwise comparisons (t-test) showed that there were no significant differences between the SONNET or the S2.Sonnet configurations and the other four SONNET 2 configurations (Fig 2).

### Speech understanding in noise (S0N0)

**Fig 3:**
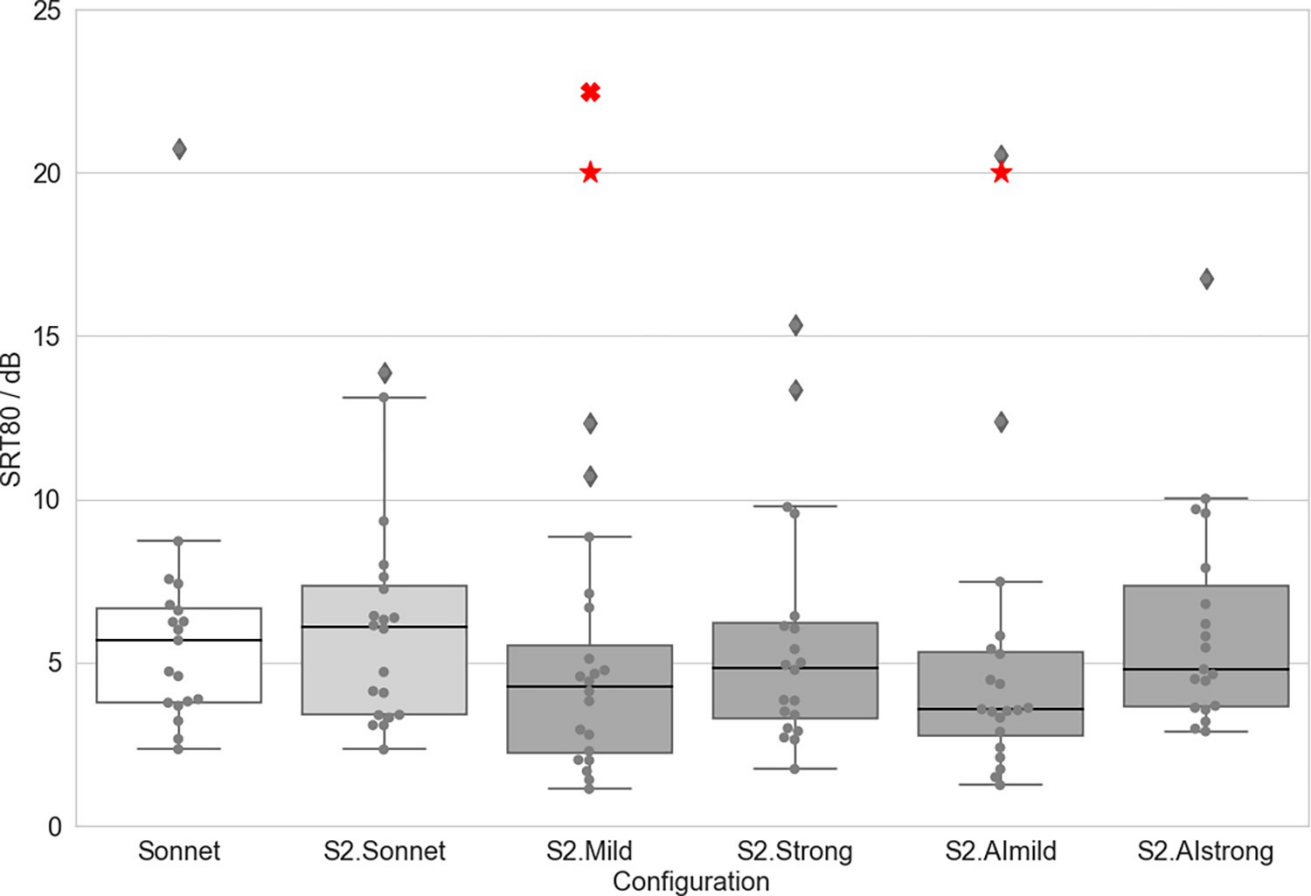
SRT80 observed in the OLSA S0N0 for each configuration. A cross marks a significant difference compared to S2.Sonnet. A star marks a significant difference compared to the SONNET. Boxplots show median values as well as first and third quartiles. Whisker bars show minimum and maximum values within 1.5 interquartile ranges below and above the first and third quartile. Diamonds show outliers.

**Fig 4:**
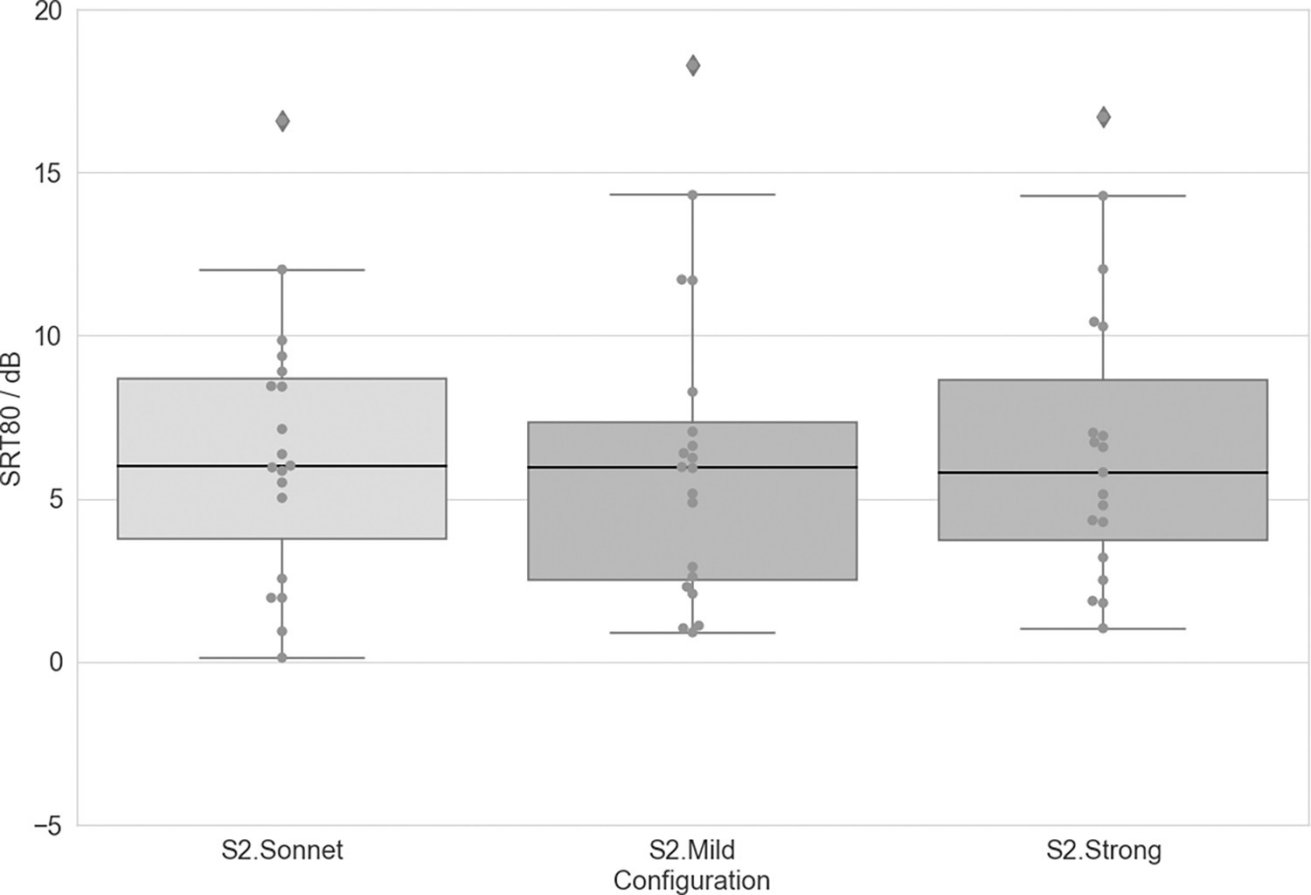
SRT80 observed in the OLSA S0N0T0 for each test configuration. Boxplots show median values as well as first and third quartiles. Whisker bars show minimum and maximum values within 1.5 interquartile ranges below and above the first and third quartile. Diamonds show outliers.

**Fig 5:**
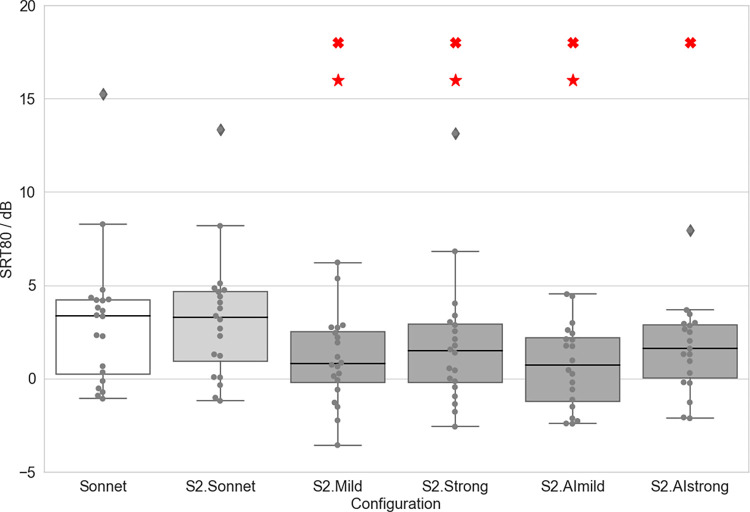
SRT80 observed in the OLSA S0N±45N±135 for each configuration. A cross marks a significant difference compared to S2.Sonnet. A star marks a significant difference compared to the SONNET. Boxplots show median values as well as first and third quartiles. Whisker bars show minimum and maximum values within 1.5 interquartile ranges below and above the first and third quartile. Diamonds show outliers.

Speech reception thresholds (SRT80) are given in Table 4 and shown in Figs 3–5 for different devices and configurations. Paired-samples t-tests revealed a significantly lower SRT (and therefore better speech perception) with S2.Mild compared to S2.Sonnet (t = 6.283, df: 19, p < 0.001). The difference between S2.Sonnet and S2.Strong was not significant, nor were the differences between S2.Sonnet and S2.AImild or S2.Sonnet and S2.AIstrong. Further, the SRT for the S2.Mild was significantly lower than for the SONNET (t = 3.44, df:18, p = 0.003). The SRT for the S2.AImild was also significantly lower than for the SONNET (t = 4.377, df:18, p < 0.001) (Fig 3).

### Speech understanding in noise (S0N0T0)

See Table 4 for scores for each tested device/configuration. Results of paired-samples t-tests showed that the three tested SONNET 2 configurations were not significantly different (Fig 4).

### Speech understanding in noise (S0N±45N±135)

See Table 4 for scores for each tested device/configuration. Paired-samples t-tests revealed significantly lower SRTs (and therefore better speech perception) compared to the S2.Sonnet configuration with both the S2.Mild configuration (t = 4.682, df: 19, p < 0.001) and the S2.Strong configuration (t = 4.236, df: 19, p < 0.001). Significantly better speech perception was also found for the SONNET 2 AI configurations (S2.AImild: t = 6.573, df: 19, p < 0.001; S2.AIstrong: t = 2.450, df: 19, p = 0.025). Significantly lower SRTs compared with the SONNET were found for the S2.Mild configuration (t = 3.885, df: 19, p = 0.001), for the S2.Strong configuration (t = 3.915, df: 19, p = 0.001), and for the S2.AImild configuration (t = 4.815, df: 19, p < 0.001) (Fig 5). Raw speech data can be found in S1 Dataset.

### Listening effort

**Fig 6:**
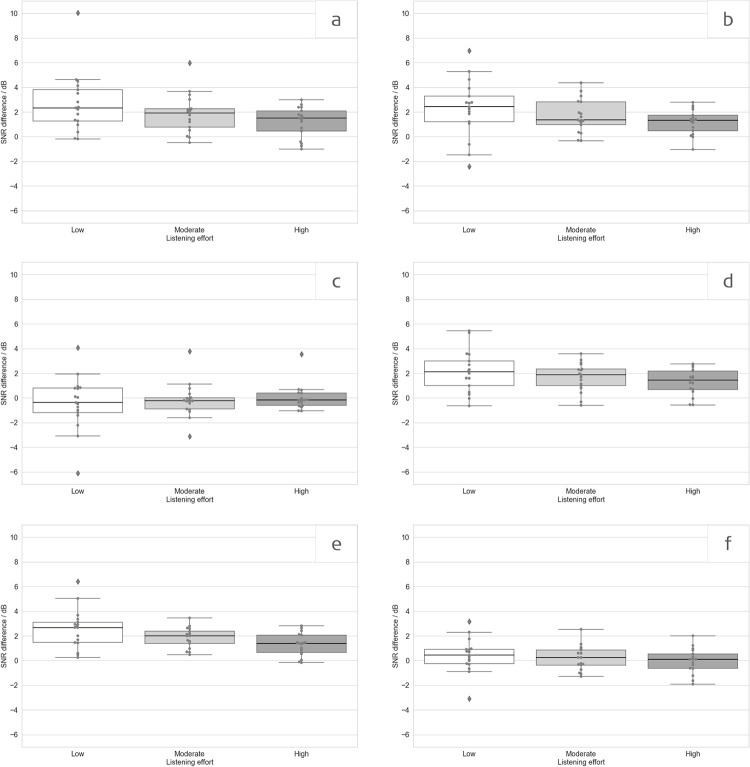
ACALES results (SNR difference, see text) for a) S2.Mild vs SONNET in S0N0, b) S2.Strong vs SONNET in S0N0, c) S2.Strong vs. S2. Mild in S0N0, d) S2.Mild vs SONNET in S0N±45N±135, e) S2.Strong vs. SONNET in S0N±45N±135, f) S2.Strong vs. S2. Mild in S0N±45N±135. Positive scores indicate a higher tolerated noise level (i.e., better performance). Boxplots show median values as well as first and third quartiles. Whisker bars show minimum and maximum values within 1.5 interquartile ranges below and above the first and third quartile. Diamonds show outliers.

Results for SNR differences between S2.Sonnet, and S2.Mild, and S2.Strong are shown in Fig 6. All differences were found to be positive, thereby confirming reduced subjective listening effort when using the SONNET 2 audio processor in configurations S2.Mild and S2.Strong (Fig 6). As stated in the Methods section, 3 subjects were excluded because their rating results did not allow a fit.

Additionally, one-sample t-tests were carried out for each effort category (low, moderate, high) to test whether the difference in dB between the S2.Sonnet and S2.Mild and S2.Strong were significantly different from zero. The differences between S2.Sonnet and S2.Mild were significantly different from zero in the S0N0 and the S0N±45N±135 test setup for all three effort categories. Likewise, the differences between the S2.Sonnet and S2.Strong were significantly different from zero in the S0N0 and the S0N±45N±135 test setup for all three effort categories (see Table 5). The differences between S2.Mild and S2.Strong were not significant. Raw ACALES data can be found in S2 Dataset.

**Table 5:** One-sample t-tests of the ACALES results.

Setup	Comparison	Effort Level	T	df	p-values (2-sided)
**S0N0**	S2.Sonnet-S2.Mild	Low	4.57	16	.000
		Moderate	4.84	16	.000
		High	3.91	16	.001
	S2.Sonnet-S2.Strong	Low	4.00	16	.001
		Moderate	5.10	16	.000
		High	4.80	16	.000
**S0N±45N±135**	S2.Sonnet-S2.Mild	Low	5.18	16	.000
		Moderate	5.96	16	.000
		High	5.11	16	.000
	S2.Sonnet-S2.Strong	Low	6.62	16	.000
		Moderate	9.33	16	.000
		High	5.79	16	.000

### Subjective sound quality and preference

Regarding preference ratings, pairwise comparisons were done across the auditory setups. No differences in the preference ratings between the S2.Sonnet configuration and S2.Mild or S2.Strong were found for speech in quiet (S0) or music in quiet (M0). For all four auditory setups with speech in noise (S0N0, S0N±45N±135, S0N0T0, and S0N±45N±135T135), the S2.Mild or S2.Strong were significantly more preferred than S2.Sonnet. Table 6 summarizes the inferential statistic results: Wilcoxon signed-rank tests (Z) or paired-samples t-tests (t), depending on the data distribution.

**Table 6:** Paired comparisons for subjects’ preference ratings between the S2.Sonnet and the SONNET 2 configurations S2.Mild or S2.Strong (original p-values).

Setup	Comparison	Test statistic	p-values (2-sided)
**S0**	S2.Sonnet–S2.Mild	Z = -.864	.387
	S2.Sonnet–S2.Strong	Z = -.189	.850
**M0**	S2.Sonnet–S2.Mild	t = -1.423	.172
	S2.Sonnet–S2.Strong	t = .298	.769
**S0N0**	S2.Sonnet–S2.Mild	t = -3.134	.006*
	S2.Sonnet–S2.Strong	t = -3.278	.004*
**S0N±45N±135**	S2.Sonnet–S2.Mild	Z = -2.614	.009*
	S2.Sonnet–S2.Strong	Z = -3.024	.002*
**S0N0T0**	S2.Sonnet–S2.Mild	Z = -3.114	.002*
	S2.Sonnet–S2.Strong	Z = -2.495	.013*
**S0N±45N±135T135**	S2.Sonnet–S2.Mild	Z = -2.688	.007*
	S2.Sonnet–S2.Strong	Z = -2.800	.005*

*Significant results after Holm-Bonferroni correction.

Concerning quality, pairwise comparisons did not show any differences between the S2.Sonnet configuration and S2.Mild or S2.Strong on sound quality and pleasantness for speech in quiet (S0) or music in quiet (M0). For all four setups with speech in noise (S0N0, S0N±45N±135, S0N0T0, and S0N±45N±135T135), S2.Mild or S2.Strong were rated with significantly higher quality for all four aspects (quality, pleasantness, listening effort, and noise disturbance) than S2.Sonnet. Table 7 summarizes the inferential statistic results: Wilcoxon signed-rank tests (Z) or a paired-samples t-tests, (t) depending on the data distribution (Table 7).

**Table 7:** Paired comparisons for subjects’ quality ratings between the SONNET and the SONNET 2 configurations S2.Mild or S2.Strong (original p-values).

Setup	Rating	Comparison	Test statistic	p-values (2-sided)
**S0**	Quality	S2.Sonnet–S2.Mild	Z = -0.241	0.809
		S2.Sonnet–S2.Strong	Z = -0.362	0.717
	Pleasantness	S2.Sonnet–S2.Mild	Z = -0.503	0.615
		S2.Sonnet–S2.Strong	Z = -1.892	0.059
**M0**	Quality	S2.Sonnet–S2.Mild	Z = -0.765	0.444
		S2.Sonnet–S2.Strong	Z = -0.241	0.809
	Pleasantness	S2.Sonnet–S2.Mild	Z = -1.248	0.212
		S2.Sonnet–S2.Strong	Z = -1.127	0.260
**S0N0**	Quality	S2.Sonnet–S2.Mild	t = -2.556	.020
		S2.Sonnet–S2.Strong	t = -2.509	.022
	Pleasantness	S2.Sonnet–S2.Mild	t = -3.684	.002*
		S2.Sonnet–S2.Strong	t = -4.816	.000*
	Listening Effort	S2.Sonnet–S2.Mild	t = -2.811	.012*
		S2.Sonnet–S2.Strong	t = -3.890	.001*
	Noise disturbance	S2.Sonnet–S2.Mild	t = -5.520	.000*
		S2.Sonnet–S2.Strong	t = -6.761	.000*
**S0N±45N±135**	Quality	S2.Sonnet–S2.Mild	Z = -2.427	.015*
		S2.Sonnet–S2.Strong	Z = -3.136	.002*
	Pleasantness	S2.Sonnet–S2.Mild	Z = -3.360	.001*
		S2.Sonnet–S2.Strong	Z = -3.584	.000*
	Listening Effort	S2.Sonnet–S2.Mild	Z = -3.360	.001*
		S2.Sonnet–S2.Strong	Z = -3.397	.001*
	Noise disturbance	S2.Sonnet–S2.Mild	Z = -3.472	.001*
		S2.Sonnet–S2.Strong	Z = -3.733	.000*
**S0N0T0**	Quality	S2.Sonnet–S2.Mild	Z = -2.199	0.028
		S2.Sonnet–S2.Strong	Z = -1.771	0.077
	Pleasantness	S2.Sonnet–S2.Mild	Z = -3.461	.001*
		S2.Sonnet–S2.Strong	Z = -3.501	.000*
	Listening Effort	S2.Sonnet–S2.Mild	Z = -3.139	.002*
		S2.Sonnet–S2.Strong	Z = -2.370	.018*
	Noise disturbance	S2.Sonnet–S2.Mild	Z = -3.702	.000*
		S2.Sonnet–S2.Strong	Z = -3.541	.000*
**S0N±45N±135T135**	Quality	S2.Sonnet–S2.Mild	t = -4.086	.001*
		S2.Sonnet–S2.Strong	t = -3.410	.003*
	Pleasantness	S2.Sonnet–S2.Mild	t = -5.607	.000*
		S2.Sonnet–S2.Strong	t = -5.906	.000*
	Listening Effort	S2.Sonnet–S2.Mild	t = -4.924	.000*
		S2.Sonnet–S2.Strong	t = -4.507	.000*
	Noise disturbance	S2.Sonnet–S2.Mild	t = -7.138	.000*
		S2.Sonnet–S2.Strong	t = -7.133	.000*

*Significant results after Holm-Bonferroni correction.

### Questionnaires

**Fig 7:**
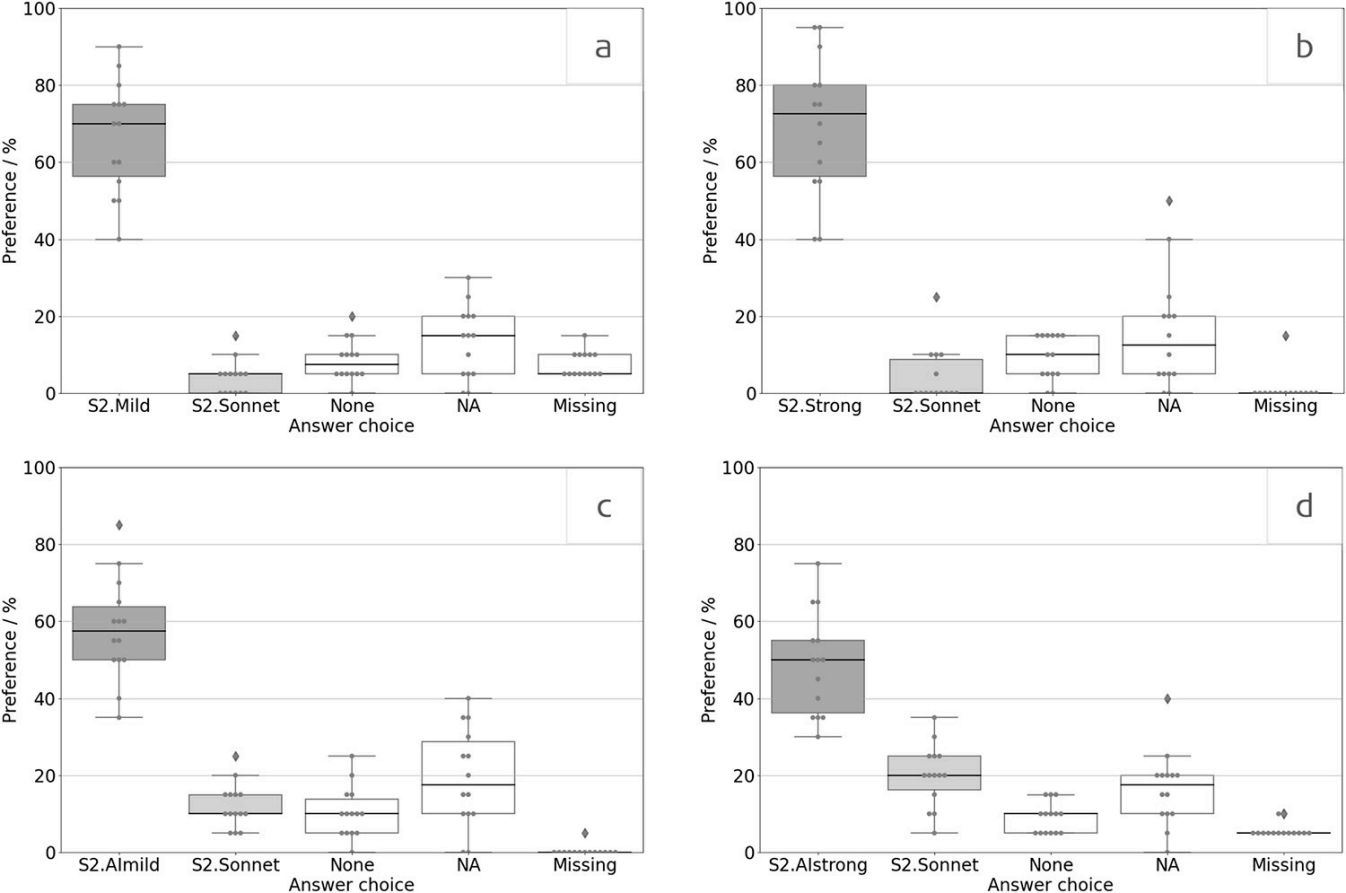
Product-specific questionnaire results. Figures are the S2.Sonnet vs. 7a) S2.Mild, 7b) S2.Strong, 7c) S2.AImild, 7d) S2.AIstrong. A point indicates that this percentage of users preferred either the SONNET 2 configuration given on the abscissa, had no preference (None), found the question not applicable (NA), or did not rate (Missing) for the specific listening environment. Higher scores indicate greater preference. Diamonds indicate outliers.

To obtain descriptive results from the product-specific questionnaire, preferences were averaged across the fourteen listening situation items in the questionnaire. Results showed a clear preference for the S2.Mild, S2.Strong, S2.AImild, and S2.AIstrong configurations over the S2.Sonnet configuration (Fig 7).

The APSQ was used to assess subjects’ satisfaction with the SONNET and the SONNET 2 audio processor using the configuration from the previous take-home period. The average APSQ total score was 8.62 (± 0.83 SD; range 7.30–9.78) for the SONNET and 8.73 (± 0.81 SD; range 7.24–9.75) for the SONNET 2. The paired t-test test did not show significant differences in subjects’ satisfaction with the two audio processors [t(19) = -.881; p = 0.389].

The HISQUI was completed at each study visit. The SONNET was rated with "moderate sound quality", while all four SONNET 2 configurations (e.g., S2.mild, S2.strong, S2.AImild, S2.AIstrong) were rated with "good sound quality". The mean HISQUI score was 85 (± 15.71 SD; range 51–110) for the SONNET and 94.74 (± 16.54 SD; range 73–132) for the S2.mild, 93.80 (± 16.41 SD; range 61–116) for the S2.strong, 93.75 (± 14.80 SD; range 57–120) for the S2.AImild, and 91.40 (± 18.49 SD; range 59–131) for the S2.AIstrong configuration, respectively. Results of the repeated measure ANOVA (N = 19) did not show a significant difference between audio processors or configurations [F(4; 72) = 1.880; p = 0.123].

The SSQ12 was completed at each study visit. Total mean scores were 5.45 (± 1.28; range 3.35–7.83) for the SONNET, 6.16 (± 1.41 SD; range 3.33–8.32) for the S2.mild, 5.53 (± 1.44 SD; range 3.42–8.11) for the S2.strong, 5.61 (± 1.15 SD; range 4.00–7.47) for the S2.AImild, and 5.34 (± 1.69 SD; range 1.75–8.40) for the S2.AIstrong configuration, respectively. The repeated-measures ANOVA (N = 13) revealed significant differences in the SSQ12 total scores between the five fitting configurations [F(4; 52) = 2.557; p = 0.049]. Paired t-tests were performed but did not show significant differences after adjusting the alpha for multiple comparisons. Raw questionnaire data can be found in S3 Dataset.

## Discussion

The primary goal of this study was to determine if the use of stationary-noise reduction (ANR), transient noise-reduction (TNR), and a signal-classification-based system named ‘Adaptive Intelligence (AI)’ would result in superior performance in listening conditions with stationary or transient noise. In short, results from speech perception tests confirmed superior performance of the SONNET 2 in stationary noise with ANR and AI but did not find an effect of TNR. Regarding subjective measures, users rated their self-perceived listening effort consistently lower with the SONNET 2 when using ANR and TNR. Likewise, they rated their subjective sound quality as higher with the SONNET 2 when using ANR and TNR in conditions with competing noise. Overall preference for SONNET 2 configurations using ANR and TNR was strong for most categories across listening with competing noise.

### Speech understanding in quiet

No significant differences were found for speech understanding in quiet, indicating that using ANR, TNR, and AI in this situation does not degrade speech perception. Since ANR and TNR were specifically designed to reduce noise in relevant conditions, it was expected that in quiet results with the SONNET 2 would be similar to those with the SONNET (or to the SONNET 2 configured as SONNET, i.e. S2.Sonnet). This result is also in line with earlier studies on noise-reduction algorithms of other CI manufacturers. Dyballa et al. [35] tested a TNR algorithm in high and low mode in an Advanced Bionics audio processor in quiet and found that speech perception was comparable to when not using TNR. The noise-reduction algorithm ClearVoice by Advanced Bionics did not compromise listening effort in quiet [19]. A Cochlear Ltd CP900 audio processor with the noise-reduction algorithm SNR-NR performed like one without noise reduction technology [36]. Similarly, the noise-reduction algorithm VoiceTrack by Oticon Medical/Neurelec did not have significant effects on speech understanding in quiet [37].

### Speech understanding in noise

#### SONNET 2 and SONNET mode (S2.Sonnet)

**I**n stationary noise, speech reception thresholds were, setups compared to the SONNET 2 in SONNET mode (S2.Sonnet), significantly better for the SONNET 2 with ANR and TNR in S0N0 (1.43 dB improvement in SRT80 for S2.Mild) and in S0N±45N±135 (2.19 dB for S2.Mild, 1.41 dB for S2.Strong, 2.55 dB for S2.AImild, 1.62 dB for S2.AIstrong). Similar significant benefits of the SONNET 2 with ANR and TNR active were observed when compared with the SONNET processor in the S0N0 setup (1.33 dB improvement in SRT for S2.Mild, 1.07 dB improvement for S2.AImild) and S0N±45N±135 (2.03 dB for S2.Mild,1.26 dB for S2.Strong, 2.4 dB for S2.AImild). In general, speech reception thresholds and improvements in SRTs in the AI configurations (S2.AImild, S2.AIstrong) corresponded well to the respective ANR/TNR configurations (S2.Mild, S2.Strong), as expected with AI activating ANR and TNR accordingly in the speech-in-noise signal class (see Table 1). Thus, in the discussion below, while we concentrate on ANR and TNR, it should be kept in mind that these features are also included in the AI switching scheme.

The benefit found for lower speech reception thresholds with an active stationary noise reduction algorithm like ANR is in line with several studies on stationary noise reduction algorithms featured in CI audio processors from various manufacturers. However, benefit varied widely across manufacturers because of different noise types, source localisation, fitting configurations, and possibly also noise reduction techniques and implementations. These parameters need to be kept in mind when comparing the results of the present study with results presented in the literature.

#### SONNET 2 and other systems

ClearVoice by Advanced Bionics is a single microphone noise reduction algorithm that aims to improve speech understanding by reducing stationary noise and emphasizing the dynamic channels that presumably contain more speech [38]. Kam et al. [39] found that subjects performed significantly better with ClearVoice in the medium setup (max. 12 dB attenuation) than with the control option (ClearVoice off) in the S0N0 setup. The average increase in speech perception in the Cantonese HINT test was a moderate 5.5%. No significant performance gain was measured for the ClearVoice high setup (max. 18 dB attenuation). Correspondingly, Koch et al. [19] found significantly improved speech understanding in speech-spectrum noise and multitalker babble, likewise in the S0N0 setup. In speech-spectrum noise, performance gains of 7.6%, 8.7%, and 10.6% for ClearVoice low, medium, and high setup, respectively, were measured using the AzBio sentence test with individually adjusted SNRs (aiming at achieving 40–50% in the control setup (ClearVoice off)). In multitalker babble noise, performance gains were smaller (ranging from 1.4% to 4.3%) and not significant. Holden et al. [40] found performance gains due to an active ClearVoice in the low, medium, and high setup only in the R-SPACE^TM^ setup (eight speaker setup with speech from 0°, and restaurant noise from the sides and behind the listener). The performance gains for this adaptive test were 1.22 dB for the ClearVoice low setup, 1.46 dB for the ClearVoice medium setup, and 2.51 dB for the ClearVoice high setup. Only the performance gain for the high setup was significant. No significant differences were found for the other administered tests, which were the BKB-SIN test (sentences from 0° and four-talker babble noise from 90° ipsilateral to the CI ear), the ANL test (acceptable noise level test), CUNY sentence test in speech-spectrum noise, and the AzBio sentences in multitalker babble noise.

Mauger et al. [20] and De Ceulaer et al. [41] evaluated the SNR-NR algorithm as implemented by Cochlear Ltd in the SmartSound iQ suite, and found a significant gain in the S0N0 setup. In Mauger et al. [20] a significant performance gain in terms of a reduced speech reception threshold due to the SNR-NR algorithm (within the SCAN option) was present only in the S0N0 condition with speech-weighted noise. The mean performance gains ranged from 1.8 dB to 2.3 dB, depending on the compared processor setups. Interestingly, no performance gains were measured either for the S0N3 condition with speech-weighted noise played from ±90° and 180° azimuth, or for the S0N3 condition with four-talker babble noise played from ±90° and 180° azimuth, or for the S0N0 condition with four-talker babble noise. In De Ceulaer et al. [41], a performance gain for SNR-NR in an S0N0 setup with speech-weighted noise of 1.2 dB (reduction of the 50% speech reception threshold, SRT50) was measured. Most of the SRT50 improvements due to SNR-NR within this study were due to subjects having an SRT50 of 5‒20 dB; thus, in a comparably high SNR range.

Guevara et al. [37] investigated the VoiceTrack noise reduction algorithm implemented in the Saphyr Neo audio processor (Oticon Medical/Neurelec) and found a significant improvement in speech perception (French disyllabic words) in speech-shaped noise (at +5 dB SNR) and cocktail party noise (at 0 dB SNR), both presented in the S0N0 setup. For the speech-shaped noise tests, significance could only be achieved when the preferred VoiceTrack setup (soft, medium, or strong) was considered. In this case, the mean improvement was 12.3%. Using a liberal least significant difference post-hoc test 1) the VoiceTrack soft setup achieved significantly better results compared to VoiceTrack off in speech-shaped noise and 2) the VoiceTrack medium setup achieved significantly better results compared to VoiceTrack off in cocktail-party noise at 0 dB SNR.

With setups that presented speech from the front and noise from spatially-separated sources, the benefit of the SONNET 2 paralleled results of previous studies. Noël-Petroff et al. [42] evaluated the ClearVoice noise-reduction algorithm in children fitted with Harmony (Advanced Bionics) audio processors and showed significant improvement for speech perception in stationary noise in an S0N135 setup. Geißler et al. [43] used ClearVoice in S0N0N±45N±90N±135N180 and S0N±70N±135N180 setups with stationary noise. Speech perception improved significantly. However, a test where roving sound was added to the second setup did not reveal any significant benefit from the use of ClearVoice. Wolfe et al. [4] tested the SNR-NR noise reduction algorithm implemented in the Nucleus 6 audio processor (Cochlear Ltd) in a S0N90 or S0N270 setup and found superior speech recognition compared to the previously used Nucleus 5 audio processor (without SNR-NR). Considering the SNR-NR algorithm of the Nucleus 6 audio processor, a mean significant improvement of 8.5% (AzBio sentence test at a fixed individually adjusted SNR) was shown (Nucleus 6 with Automatic sensitivity control (ASC) + adaptive dynamic range optimization (ADRO) and standard microphone mode, SNR-NR disabled vs. Nucleus 6 with ASC + ADRO and SNR-NR enabled with standard microphone mode)).

Regarding transient noise reduction, speech recognition was comparable between SONNET 2 in SONNET configuration (S2.Sonnet, no noise reduction) and the SONNET 2 with ANR/TNR (S2.Mild, S2.Strong). This result can be explained by the specific type of transient noise used in this study. Although hammering usually dominates speech signals in terms of level, it is very short temporally. Thus, with this noise signal, enough speech components remained so that word recognition was similar between the non-TNR (S2.Sonnet) and the two TNR conditions (S2.Mild, S2.Strong), with SRT values being comparable. At the same time, TNR perceptively reduced the amplitude of the transient noise signal, which consequently led to the superior subjective preference and quality ratings found for S2.Mild, S2.Strong.

Unfortunately, to the best of our knowledge few studies have investigated transient noise reduction in CI systems. This is likely due to only one other manufacturer (Advanced Bionics) offering a TNR algorithm. Dyballa et al. [35, 44] tested a TNR by Advanced Bionics and found significant improvements in SNRs when using single- or multiband TNR algorithms. However, the sound tokens (e.g. speech and hammering noise) were delivered directly via an input jacket to the audio processor. In Dingemanse et al. [21], the TNR algorithm SoundRelax was run on a Q70 audio processor (Advanced Bionics) in the S0N0T0 setup with recorded transient kitchen sounds (clinking bowls, dishes, etc.). Interestingly, and in contrast to the results found here, TNR could not prevent transient noise lowering speech understanding in noise, although it did reduce subjective annoyance from transient sounds.

In summary, the results for ANR and TNR in the SONNET 2 sound processor show significant gains in speech perception in stationary noise and compare well with similar technologies in other CI sound processors. These gains exist regardless of whether ANR and TNR are explicitly activated in the SONNET 2 processor (S2.Mild, S2 Strong) or are used as part of AI (S2.AImild, S2.AIstrong). In combined stationary and transient noise, ANR and TNR do not lead to a decrement in speech perception, which at least means that the benefits in subjective sound quality from these technologies (as discussed below) come without any penalty in speech perception.

### Listening effort, subjective sound quality and preference, questionnaires

Like in the previous section, the discussion below will concentrate on ANR and TNR. It should be kept in mind that these features are also included in the AI switching scheme.

Two different tests assessed listening effort and perceived sound quality in this study. The ACALES test quantified perceived listening effort for speech perception in noise. Its results were in line with those found for the objective speech understanding in noise tests: subjects tolerated consistently lower SNR values with the SONNET 2 configurations (S2.Mild, S2.Strong) compared to the SONNET configuration (S2.Sonnet) in all hearing effort categories. In fact, the speech-in-noise tests revealed that the objective benefit in speech understanding in noise was consistent with the subjective ratings. More precisely, there was a 1–2 dB advantage for the SONNET 2 configurations S2.Mild and S2.Strong in both setups, S0N0 and S0N±45N±135, throughout all three effort categories, e.g. low, moderate, and high effort. Consistently, the speech-in-noise results also showed a similar advantage for these two SONNET 2 configurations (only the advantage in the ANR/TNR strong configuration in the S0N0 condition was not significant). Thus, the subjects’ subjective self-assessment of their perceived hearing effort closely reflected the improvements in SRT. We therefore conclude that, users not only tolerate a smaller SNR with a noise reduction algorithm in place, but this noise reduction algorithm improves speech understanding in noise, that is, it effectively lowers the SRT.

#### SONNET 2 and other systems

The above findings are in contrast with those described for the ClearVoice algorithm of Advanced Bionics. Dingemanse & Goedegebure [45] used the acceptable noise level (ANL) test [46] in a similar cohort of CI recipients and found a significant improvement, i.e., higher background noise levels were tolerated. However, they did not find any corresponding improvements in speech understanding in background noise, in any of the determined SRTs (SRT50, SRT70) or at a fixed SNR (SRT50 + 11dB). Differently to our study where the SRT80 was determined, they determined the SRT50 or SRT70. While these results might highlight differences between ClearVoice and ANR as well as TNR, one needs to also consider differences between the ACALES and the ANL test. Although similar in nature, they may differ in their specificity. The ACALES test might be better suited to self-assess the speech understanding in noise because the subjects must relate their hearing effort during the test directly to their ability to understand speech in noise. With the ANL test, subjects are asked for their personally acceptable background noise level, which might be less related to their ability to still understand speech but more to their overall hearing experience (e.g. their “willingness to listen to speech in background noise”, cf. Dingemanse & Goedegebure [45]). A further difference between the two tests is that the ACALES used in the present study employed the same speech (and noise) signals as the objective speech understanding tests (OLSA sentences and OLSA speech-shaped noise). In Dingemanse & Goedegebure [45], the speech and noise material used and speech understanding tests were different in that for the ANL a recorded story in noise was administered, whereas unrelated sentences of female speech of five to nine words were used in the objective speech in noise test (a proprietary test developed by the authors).

The other subjective assessments of the present study were the preference and quality ratings of various test signals. Again, these results were in line with those of the objective speech perception tests. Firstly, the ANR and TNR were both generally found to not affect speech understanding or music perception in quiet. Thus, there was no perceived negative effect of an active ANR and TNR. This parallels the outcomes of the Freiburg monosyllabic test, administered in quiet, which showed no difference in monosyllable perception for the SONNET 2 when using ANR and TNR (S2.Mild, S2.Strong, S2.AImild, S2.AIstrong) in comparison to tests without ANR and TNR (SONNET, S2.Sonnet). This is a remarkable finding, since music signals typically contain transient components, which are left intact by the active TNR. Secondly, the SONNET 2 configurations S2.Mild and S2.Strong were consistently preferred and better rated for speech in stationary and/or transient noise. Most notably, the hearing effort and the perceived disturbance by stationary and transient noise were rated lower for the SONNET 2 configurations S2.Mild and S2.Strong. Also, the sound quality and pleasantness were graded as being better in both SONNET 2 configurations. This indicates that the reduction of the stationary and transient disturbance by an active ANR and TNR led to no perceivable loss in sound quality.

Dingemanse et al. [21] showed that the simultaneous activation of the stationary-noise reduction ClearVoice and the transient noise reduction SoundRelax by Advanced Bionics did not result in subjective improvement when comparing various sounds contaminated with stationary or transient noise. This was perhaps due to a 5% increase of M-levels when ClearVoice was active, which in turn might have led to an increased loudness perception of transient noise. Although this negative effect was corrected by activating the transient noise reduction algorithm (SoundRelax), subjects only took advantage of this functionality when ClearVoice was not active. Thus, there was a negative effect of ClearVoice in environments with transient noise signals present. Such an effect was not seen in the SONNET 2 when both ANR and TNR were activated, since both configurations that use ANR and TNR (S2.Mild, S2.Strong) were 1) preferred over the SONNET configuration (S2.Sonnet, i.e., ANR and TNR off) and 2) rated better for all presented test signals, irrespective of the presence of stationary and/or transient noise. This was also reflected in the results of the product-specific questionnaire, where subjects strongly and consistently rated all SONNET 2 configurations higher than the SONNET configuration across various listening situations. In addition, the questionnaire proved to be a relevant assessment of the AI modes (S2.AImild, S2.AIstrong) in that they showed that AI, like the configurations using ANR and TNR, was preferred over SONNET for fourteen real-life auditory scenarios. APSQ scores did not differ between SONNET and SONNET 2, but nonetheless, subjects rated their satisfaction with both processors as very high. For the HISQUI, the difference between the SONNET configuration and the four SONNET 2 configurations also reflected different sound quality levels, with moderate sound quality for the SONNET and good sound quality for the SONNET 2 configurations. Finally, the SSQ12 did not show any significant differences between the five configurations after alpha-correction. Nonetheless, these results were descriptively higher than those in a recent publication [47].

## Conclusions

The MED-EL SONNET 2 audio processor features new front-end functionalities: ambient noise reduction (ANR); transient noise reduction (TNR); and Adaptive Intelligence (AI), which is a classifier-based automated program-switching scheme. All three technologies resulted in significant improvements in speech understanding in noise in a variety of different test setups. This was paralleled by significantly reduced perceived listening effort when using these functionalities as well as a strong subjective preference of these functionalities in real-life auditory scenarios.

## Supporting information

S1 Table Table of acronyms. (DOCX)

S1 Dataset Speech test data. (XLSX)

S2 Dataset ACALES data. (XLSX)

S3 Dataset Questionnaires data. (XLSM)
