# Supplementary material for: Improved performance with automatic sound management 3 in the MED-EL SONNET 2 cochlear implant audio processor
Source: PLoS One. 2022 Sep 15;17(9):e0274446. doi: 10.1371/journal.pone.0274446 (PMC9477286; doi:10.1371/journal.pone.0274446)
Supplement: S1 Table — (DOCX) [file pone.0274446.s001.docx]

Table of acronyms (in alphabetical order)

| ABF | Adaptive beamformer |
| --- | --- |
| ACALES | Adaptive Categorical Listening Effort Scaling |
| ADRO | Adaptive dynamic range optimization |
| AGC | Automatic gain control |
| AI | Adaptive intelligence |
| ANL | Acceptable noise level test |
| ANR | Ambient noise reduction |
| APSQ | Audio Processor Satisfaction Questionnaire |
| ASC | Automatic sensitivity control |
| CI | Cochlear implant |
| HISQUI | Hearing Implant Sound Quality Index |
| NAT | fixed (‘natural’) beamformer |
| OLSA | Oldenburg Sentence Test |
| OMNI | Omnidirectional beamformer |
| PTA | Pure tone average |
| SNR | Signal-to-noise ratio |
| SPL | Sound pressure level |
| SRT | Speech reception threshold |
| SSQ12 | Speech, Spatial and Qualities of Hearing scale |
| TNR | Transient-noise reduction |
| WNR | Wind-noise reduction |
